# Ultrasound-Guided Regional Anesthesia in Permanent Pacemaker Implantation: An Observational Study

**DOI:** 10.3390/medicina61061001

**Published:** 2025-05-28

**Authors:** Hakan Akelma, Enes Çelik, Yusuf İpek, Mehmet Ali Turgut, Muhammed Raşit Tanırcan, Adem Aktan, Mehmet Zülküf Karahan

**Affiliations:** 1 Department of Anesthesiology and Reanimation, Mardin Artuklu University, Mardin 47100, Turkey; anestezistenescelik@gmail.com; 2 Department of Anesthesiology and Reanimation, Mardin Training and Research Hospital, Mardin 47100, Turkey; dr.y.ipek40@gmail.com (Y.İ.); turgutmali3@gmail.com (M.A.T.); 3 Department of Cardiology, Mardin Training and Research Hospital, Mardin 47100, Turkey; drtanircan21@hotmail.com; 4 Department of Cardiology, School of Medicine, Mardin Artuklu University, Mardin 47100, Turkey; dradem21@hotmail.com (A.A.); zerkif75@gmail.com (M.Z.K.)

**Keywords:** local anesthesia, pacemaker implantation, pain management, peripheral nerve blocks, regional anesthesia

## Abstract

*Background and Objectives:* When pacemakers were first introduced, their indications and implantation techniques were quite limited. Over 400,000 permanent pacemakers are implanted annually worldwide, mostly under local anesthesia (LA), which is preferred for its hemodynamic stability. However, inadequate LA often leads to excessive use of local anesthetics or analgesics. This study evaluates the efficacy of combining interscalene brachial plexus block (ISB) and superficial cervical plexus block (SCPB) as regional anesthesia (RA) techniques during permanent pacemaker implantation compared to LA. *Materials and Methods:* A total of 42 patients were divided into RA and LA groups. The RA group underwent ISB and SCPB under ultrasound guidance, while the LA group received traditional local anesthetic methods. *Results:* The RA group exhibited superior pain control, reduced analgesic requirements, and higher satisfaction rates compared to the LA group. Ultrasound guidance enhanced block success rates and minimized complications. *Conclusions:* ISB and SCPB offer a superior alternative to LA for pacemaker implantation, especially in patients with anxiety or insufficient LA response.

## 1. Introduction

The initial applications of pacemakers were limited in terms of both indications and implantation techniques. Today, over 400,000 permanent pacemakers are implanted annually worldwide. Technological advancements have reduced the invasiveness of these procedures, thereby shifting the responsibility from surgeons to cardiologists [1,2,3].

Local anesthesia (LA) is widely used during permanent pacemaker implantation, particularly in elderly patients and those with cardiac dysfunction. It offers a significant advantage in maintaining hemodynamic stability. However, inadequate anesthesia often results in excessive use of local anesthetics or supplemental analgesics [4].

In elderly patients with cardiac disease, the need for intravenous anesthetics can often be avoided through peripheral nerve block techniques. In cases where local anesthesia proves insufficient or patient anxiety is pronounced, regional anesthesia (RA) combined with sedation is preferred. Compared to general anesthesia (GA) or sedation, RA demonstrates a lower risk of perioperative morbidity and mortality in elderly populations and patients with cardiac dysfunction [4,5].

Upper extremity nerve blocks offer an effective RA option for the innervation of the clavicle, chest wall, and overlying skin. Techniques such as interscalene block (ISB), supraclavicular block, superficial cervical plexus block (SCPB), paravertebral block, dorsal scapular and suprascapular nerve blocks, intercostal nerve blocks, and pectoral nerve (PECs) blocks can be safely performed under ultrasound (USG) guidance [6].

The use of USG guidance for identifying nerves in the cervical region is well documented in the literature [7]. This imaging technique enhances safety by enabling precise visualization of anatomical structures and reducing the required doses of local anesthetics. Furthermore, the USG provides a valuable aid in the observation of anesthetic dispersion around the brachial plexus, offers support for supplementary injections, and has been shown to improve block success rates [8].

After transvenous pacemaker implantation, the chest wall’s dense innervation frequently causes tissue inflammation, thereby increasing the need for effective analgesia. Providing adequate pain management in both the preoperative and postoperative periods is essential for early recovery, reducing postoperative complications, and supporting early mobilization [9].

This study aims to evaluate the efficacy of ISB combined with SCPB compared to local anesthesia in patients undergoing permanent pacemaker implantation.

## 2. Materials and Methods

### 2.1. Patient Selection and Study Design

This study was approved by The Ethics Committee of Mardin Artuklu University on 21 December 2022 (approval no. 78079) and was conducted in accordance with the principles outlined in the Declaration of Helsinki. Patients treated during the following six months were included, and written informed consent was obtained from all participants.

This prospective observational study included patients who visited the cardiology outpatient clinic and were scheduled for permanent pacemaker implantation between January and April 2023. Initially, 53 patients were enrolled. However, five patients from the regional anesthesia (RA) group declined the procedure and were excluded from the study as they did not meet the criteria for transfer to the local anesthesia (LA) group. Similarly, six patients from the LA group were excluded: three refused the procedure, and three required rescheduling due to unsuccessful venous access. The final analysis included 42 patients, with 21 in each group.

Inclusion Criteria: Patients between 18 and 85 years of age who were suitable for permanent pacemaker implantation.

Exclusion Criteria: Patients who refused the procedure or who had significant hepatic or renal disease, infection at the site of the procedure, allergy to local anesthetics or opioids, severe coagulopathy, advanced pulmonary disease, a history of cardiothoracic surgery, chest wall deformities, or rib fractures were excluded.

### 2.2. Randomization and Blinding

Before the procedure, all patients received training in pain assessment using the Visual Analog Scale (VAS) and Numerical Rating Scale (NRS). Following anesthesia application (RA or LA), sensory block adequacy was evaluated using pin-prick and cold sensation tests. For patients receiving interscalene block (ISB), limb muscle strength was assessed using the MRC Muscle Power Scale (no contraction = 1; normal strength = 5) (Table 1). Pain levels were measured independently by a separate anesthesiologist using the VAS and NRS (no pain = 0; worst pain possible = 9–10).

Patients were randomly assigned to either the RA or LA groups via sealed envelopes containing numbers (1 for RA, 2 for LA). Patients, blinded to the method of anesthesia, were referred to the angiography unit. RA procedures were performed by a single anesthesiologist, while LA was administered by a team of cardiologists. In the RA group, no additional local anesthetics were administered.

## 3. Objective and Aim

**Figure 1:**
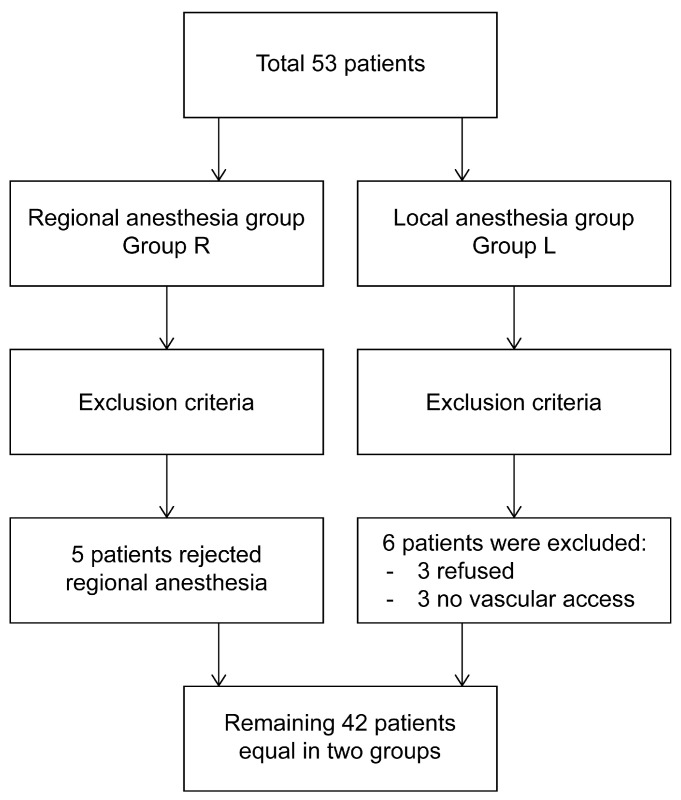
Flowchart.

**Figure 2:**
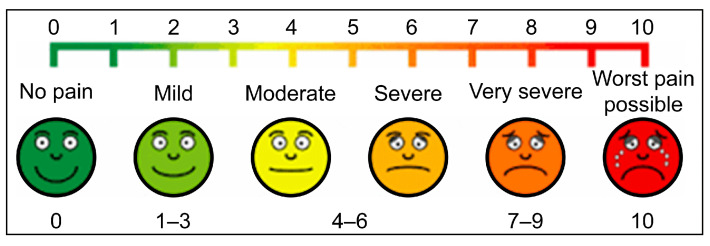
Visual Analog and Numeric Scale.

In designing this study, which aims to compare the efficacy of RA and LA for pacemaker implantation, we focused on two key aspects (Figure 1 and Figure 2).

**Figure 3:**
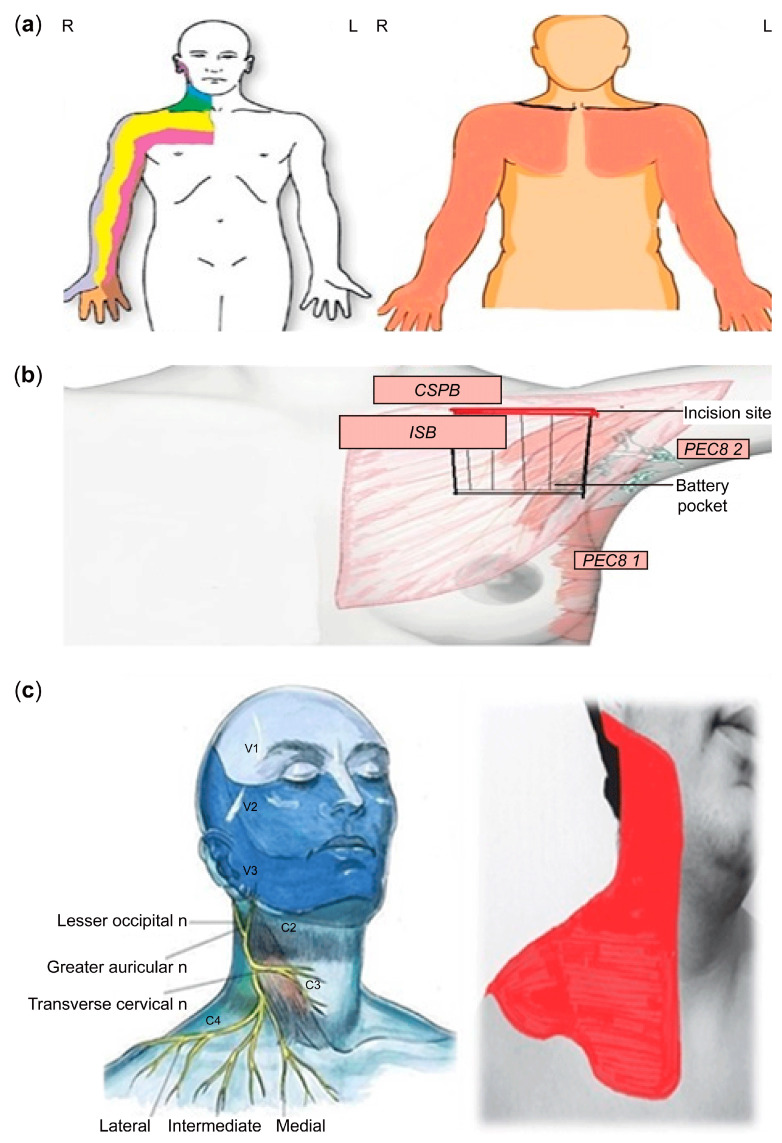
(**a**) Brachial nerve innervation areas, (**b**) the pocket part to be prepared for the battery compartment and the anatomical areas of the upper and lower clavicle (proximal pectoral region), and (**c**) superficial cervical plexus distribution.

First, we investigated the sensory innervation of the procedural site. The anatomical region where venous access will be obtained and the pacemaker pocket will be created primarily involves the superior and inferior parts of the clavicle (proximal pectoral region) rather than the shoulders and arms. This area is innervated both superficially and deeply by various nerve plexuses (Figure 3).

**Figure 4:**
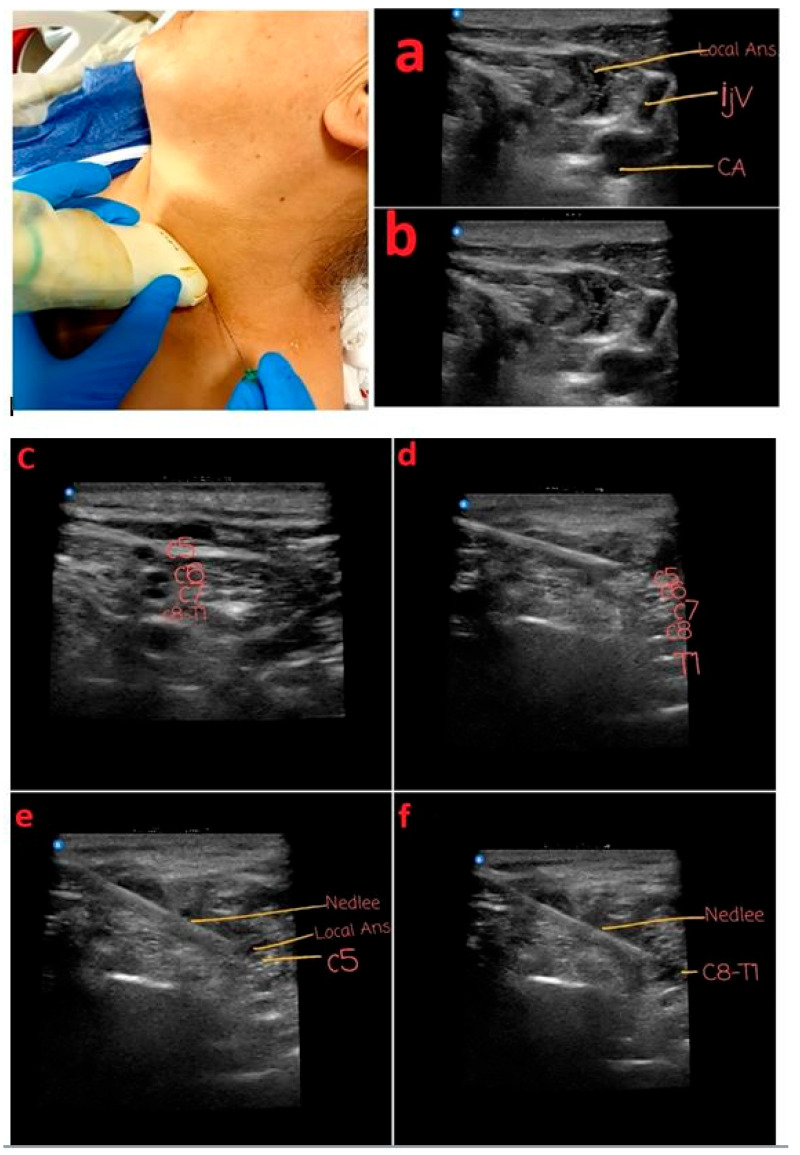
(**a**,**b**) Superficial cervical plexus block technique. Local Ans: **local anesthetic distribution**, IJV: internal jugular vein, and CA: carotid artery. Interscalene block with USG: targeting and blocking the C5 and C8–T1 roots. (**c**) Interscalene nerve alignment. (**d**) Interscalene nerve and puncture needle. (**e**) Targeting the C5 nerve root with the puncture needle. (**f**) Targeting the T1 nerve root with the puncture needle.

The relevant nerves include the interscalene plexus (C5–T1 roots), which is primarily responsible for deep innervation. The superficial cervical plexus (C3–C4 supraclavicular branches) provides mostly superficial innervation through its lateral, medial, and intermediate branches. Additionally, the pectoral nerves, intercostal nerves, lateral cutaneous branches of the long thoracic and thoracodorsal nerves, and subpectoral nerves contribute to both superficial and deep innervation (Figure 4).

In order to achieve the objective of blocking both the deep and superficial regions, it was decided that a combination of an interscalene block (ISB) and a superficial cervical plexus block (SCPB) would be the most appropriate approach.

Our second objective was to determine the optimal dose of anesthetic drugs. To achieve this, we aimed to avoid a complete motor block during ISB. A motor block can result in an arm drop on the left side, which may cause discomfort for the patient. Therefore, we diluted the local anesthetic dose to provide a deep sensory block while minimizing the motor block. The anesthetic drug dosage was determined as follows: 60 mg lidocaine (3 mL of 2%) + 50 mcg fentanyl (1 mL) + 16 mL saline (SF). This mixture was prepared to achieve a total volume of 20 mL.

## 4. Anesthesia Preparation

All patients underwent routine preoperative anesthesia evaluations.

Under ultrasound guidance, a superficial cervical plexus block (SCPB) was performed by imaging at the midpoint of the sternocleidomastoid (SCM) muscle. Ten milliliters of 2% prilocaine was infiltrated beneath the SCM fascia. Similarly, an interscalene block was performed under ultrasound guidance by identifying the brachial plexus at a depth of 1–3 cm. A block needle was positioned within the tissue space between the anterior and middle scalene muscles, and local anesthetic was injected until the spread around the brachial plexus was visualized on ultrasound. In this procedure, which primarily targets the C5 and T1 nerve roots, a total of 20 mL of solution was administered, consisting of 60 mg lidocaine (2%, 3 mL) + 50 mcg fentanyl (1 mL) + 16 mL saline (SF). When the T1 root could not be visualized, a local anesthetic was applied to the distal portions of the C7 and C8 roots.

**Cold Test and Pin-Prick Test:** Pain sensation using a sterile needle and cold sensation using an ice pack was assessed at 5, 10, and 15 min after the block.

### 4.1. Fluoroscopic-Guided Vascular Puncture in Posterior–Anterior (PA) Projection

**Figure 5:**
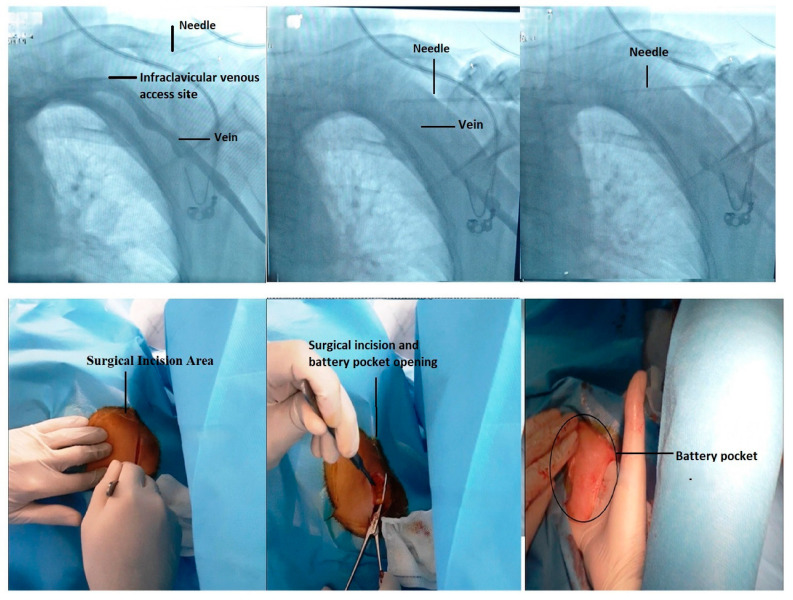
Pacemaker venous puncture technique and surgical technique.

In this study, the fluoroscopic-guided vascular puncture technique in the posterior–anterior projection, commonly used in our cardiology clinic, was employed. The area was prepared under aseptic conditions, and after traversing the skin and subcutaneous tissues, a pacemaker pocket was created. The venous puncture was performed under fluoroscopic guidance (Figure 5). The needle used for puncture was directed at an angle of approximately 60–70° toward the intersection region between the clavicle and the first rib. Verification was achieved through venous blood aspiration, and the puncture was completed with the assistance of contrast-enhanced venography.

### 4.2. Postoperative Procedure

All patients were monitored in the cardiology intensive care unit for at least 30 min following the procedure. During this period, the postoperative patient satisfaction (PPS) survey was administered; the results of which were recorded. This survey was developed by Kiyak et al. to assess patient satisfaction in relation to surgical outcomes [11]. The questionnaire employs a 7-point Likert scale to enable respondents to rate their level of satisfaction with each question [12] (Table 2 and Table 3).

Patients were observed in the hospital for one day, during which they were monitored for hematoma, swelling, and edema. A follow-up appointment was scheduled for discharged patients one week later. This appointment was intended to remove sutures, assess wound healing, and check for signs of infection.

## 5. Statistical Methods

Data analysis was performed using SPSS version 26 (SPSS Inc., Chicago, IL, USA). Categorical data were presented as frequencies and percentages (%), while quantitative data were summarized using mean and standard deviation (SD) for normally distributed data and median and range for non-normally distributed data.

For categorical variables, the chi-square test (Fisher’s exact test) was used to compare two groups. For normally distributed quantitative variables, the independent samples *t*-test was employed, while the Mann–Whitney U test was used for non-normally distributed data. A *p*-value < 0.05 was considered statistically significant.

## 6. Results

A total of 42 patients meeting the inclusion criteria were divided into two groups: the local anesthesia group (Group L) and the regional anesthesia group (Group R). No statistically significant differences were found between the two groups in terms of demographic characteristics, procedure duration, or the need for additional doses of anesthetic agents (Table 4). The power of the study was calculated using the G*Power 3.1.9.7 program. In the calculation, the effect size (d) for the *t*-test in two independent samples consisting of equal samples (n1 = n2 = 21) was determined as 0.8, and the power of the study was calculated as 0.86 [13].

Our assessments revealed that the VAS and muscle strength examination (MSE) scores were significantly higher in Group L compared to Group R (*p* < 0.001). Patients in the regional anesthesia group reported significantly higher scores across all items of the surgical satisfaction questionnaire (SSQ) than those in the local anesthesia group (*p* < 0.001).

In the pin-prick test analysis, the absence of block formation at 5 min was significantly more frequent in Group L (*p* < 0.004). However, this difference was not statistically significant at subsequent time intervals.

In the cold sensation test, Group L demonstrated significantly lower anesthesia effectiveness at all time points (*p*-values: <0.001, <0.017, and <0.035, respectively). These findings suggest that regional anesthesia provides a faster and more effective sensory block compared to local anesthesia.

## 7. Discussion

In our study evaluating anesthesia methods in patients undergoing pacemaker implantation, the regional anesthesia group showed superior outcomes compared to the local anesthesia group in terms of VAS scores, PPS results, and post-surgical patient satisfaction questionnaire (PSPSQ) answers.

Pacemaker implantation is often performed on patients who are elderly and frail. In this vulnerable patient group, local anesthesia alone is frequently used to avoid opioid administration and maintain hemodynamic stability. Regional anesthesia techniques have been highlighted as a good alternative to general anesthesia, especially in high-risk patients with comorbidities such as COPD or Duchenne muscular dystrophy (DMD) [14]. Currently, there is no standard method, drug dosage, or composition defined in the literature for pacemaker implantation [15]. Central blocks, such as thoracic epidural analgesia, are avoided in this patient group because they may cause sympathetic blockade. Other methods include general anesthesia (GA), regional anesthesia (RA), local anesthesia (LA), sedation–analgesia, and monitored anesthesia care (MAC) [16]. It is important to note the potential risks associated with GA, including the possibility of hypotension and bradycardia. Conversely, MAC may increase the likelihood of airway obstruction.

Several studies have pointed out that pain during pacemaker implantation is often underestimated and inadequately treated [17,18]. Bode et al. emphasized that inadequate pain management occurs in approximately 60% of cases during pacemaker implantation [19]. Insufficient analgesia and the resulting stress may lead to complications such as stress cardiomyopathy and complex regional pain syndrome [20,21]. Studies conducted in the United States have highlighted the excessive use of postoperative opioids and recommended the use of peripheral nerve blocks to reduce opioid consumption. It has been suggested that pain management during and after the procedure should be optimized to minimize opioid use [22,23].

Various regional anesthesia techniques, including thoracic paravertebral block, PECs I and II blocks, serratus anterior plane block, interscalene block, and cervical plexus blocks, have been utilized [16]. However, in the past, some centers have preferred using GA, assuming it provided more stable hemodynamic conditions [24]. The use of GA, however, not only increases costs but also prolongs procedure times. Additionally, there is concern about its potential impact on left ventricular function in elderly patients. Kaya et al. highlighted the risks of cardiovascular depression associated with the use of propofol for GA in high-risk patients and proposed conscious sedation as an alternative method [25]. However, their study differed from ours, as it included the use of intravenous fentanyl and did not assess postoperative pain.

There are few studies in the literature evaluating the use of RA during pacemaker implantation [26]. While planning our study, we referred to research on breast surgery, port placement, and clavicular surgery due to the overlapping procedural sites. Given the absence of a standardized approach, as highlighted by Çevikalp and Yapıcı, and the shared sensory innervation of the clavicle by the cervical and brachial plexuses, we selected a combined approach of interscalene block (ISB) and superficial cervical plexus block (SCPB) [27].

Raza et al. achieved adequate anesthesia by combining SCPB with T2, T3, and T4 intercostal nerve blocks [28]. Fujiwara et al. provided sufficient analgesia for CRT device implantation by using PECs and intercostal nerve blocks [29]. Mavarez et al. reported effective anesthesia for pacemaker implantation using PECs II block combined with local anesthesia [30]. Both studies utilized PECs blocks; however, Fujiwara’s study included dexmedetomidine infusion, while Mavarez supplemented PECs block with a local anesthetic at the surgical site. These case reports differ from the findings of our study, as we avoided the use of IV analgesic drugs and local anesthesia in the regional group to ensure more definitive results. We believe that the use of dexmedetomidine infusion and local anesthesia in these studies may have influenced their outcomes.

Additionally, we believe that ISB and SCPB offer superior anesthesia for pacemaker implantation compared to PECs blocks. Our study demonstrated that the use of multiple blocks or anesthesia methods in these studies further reinforces the need for combining blocks.

Tran et al. highlighted the debated nature of clavicular innervation, identifying supraclavicular, subclavian, long thoracic, and suprascapular nerves as contributors [31]. Similarly, Balaban et al. observed that the optimal approach for clavicular surgery remains a topic of ongoing debate. They advocated for the combined use of ISB and SCPB for effective anesthesia. Clavicular skin innervation has not been clearly defined in the literature, with sources varying between C3 and C6 [32]. A review on RA in clavicular fractures and surgery emphasized that sufficient anesthesia cannot be achieved with a single peripheral nerve block due to the complex innervation of the clavicle. Better outcomes have been reported when combining ISB with superficial or intermediate cervical plexus block [33].

A multicenter retrospective study on RA for clavicular surgery compared patients who received ISB and SCPB to those who received SCPB alone. The combination group demonstrated lower pain scores and reduced opioid requirements [34]. Similarly, Abu Sabaa et al. and Kukreja et al. emphasized the dual innervation of the clavicle from the cervical and brachial plexuses, recommending the blockade of both plexuses for effective anesthesia in clavicular surgery [35,36]. In light of these studies, we believe the combination of ISB and SCPB is an appropriate technique for our study.

Additionally, instead of the traditional approach, we used ultrasound (USG)-guided single-shot ISB, aiming to specifically target the C5 and T1 nerve roots. We achieved better sensory block and surgical comfort by including C5 in the block. Care was taken to avoid motor block in the arm while ensuring effective sensory block at the procedural site. This approach also facilitated the preservation of diaphragm function, which was a significant advantage. Although we aimed to target C5 and T1 separately, we observed that the local anesthetic spread within the same sheath, inevitably affecting other nerves in the sheath.

Preoperative initiation of pain management has been recommended in many studies to enhance postoperative pain control. Inadequate analgesia during the procedure may lead to a frozen shoulder due to restricted arm movement caused by postoperative pain anxiety [23]. Akhondzadeh et al. and Prasad et al. reported that the use of fentanyl as an adjuvant in peripheral nerve blocks enhanced block efficacy and prolonged its duration [37,38].

Meta-analyses of epidural anesthesia during labor have compared high- and low-concentration epidural local anesthetics. The results of these studies indicated that low-concentration drugs resulted in a reduction in the incidence of side effects and motor blockade while maintaining the quality of the analgesia [39]. Similarly, the use of low-concentration local anesthetics with fentanyl for labor epidurals was noted to reduce motor blockade and hypotension [40,41,42].

In our study, we aimed to provide analgesia without causing motor blockade to prevent postoperative arm immobility and the anxiety associated with the sensation of paralysis during the procedure. Strong local anesthetics such as bupivacaine were avoided. Instead, a low-concentration lidocaine mixture was used, which was supplemented with fentanyl to enhance block efficacy and duration.

According to the results of the pin-prick and cold tests, the regional anesthesia group demonstrated a faster onset of action compared to the local anesthesia group. Additionally, the regional anesthesia group exhibited lower VAS scores, a reduced need for supplemental analgesics, and fewer patient complaints related to motor blockade. The surgical satisfaction survey indicated that operator satisfaction was also higher with regional anesthesia compared to that with local anesthesia.

## 8. Study Limitations

The main limitations of our study include its single-center design and small sample size. Furthermore, the lack of long-term follow-up limited the evaluation of postoperative analgesic efficacy. Larger-scale, multicenter studies are needed to address these limitations.

## 9. Conclusions

We believe that the combination of ISB and SCPB is one of the most effective methods for peripheral nerve block management in pacemaker implantation. This technique offers significant advantages, including reduced postoperative analgesic use, early upper extremity mobility, enhanced surgical and patient comfort, rapid and effective anesthesia, and maintained hemodynamic stability. Our findings suggest that this approach provides better pain management and patient satisfaction compared to local anesthesia during both the intraoperative and postoperative periods.

## Figures and Tables

**Table 1:** MRC Muscle Power Scale [10].

Score	Description
0	No contraction
1	Flicker or trace of contraction
2	Active movement, with gravity eliminated
3	Active movement against gravity
4	Active movement against gravity and resistance
5	Normal power

**Table 2:** Post-surgical patient satisfaction survey (PSTQ).

Post-Treatment Questionnaire Please Circle Your Response to Each Question							
Question	1. Not at All	2	3	4. Neutral	5	6	7. Very Likely
If you had to make the decision again, how likely would you be to undergo this same surgery?							
How likely would you be to recommend this same surgery to others?							
Considering everything, how satisfied are you now with the results of surgery?							
Overall, how satisfied are you with your current bite?							
Overall, how satisfied are you with your current speech articulation?							
Overall, how satisfied are you with your current lip posture and lip closure?							
Overall, how satisfied are you with your current breathing?							
Overall, how accepting are you with your current level of TMJ/facial pain?							
Overall, how accepting are you with your current level of lower lip/chin sensation?							

**Table 3:** The 7-point Likert scale.

1	I strongly disagree
**2**	I disagree
**3**	I partially disagree
**4**	Neither agree nor disagree
**5**	Partially agree
**6**	Agree
**7**	Strongly agree

**Table 4:** Statistical data.

Measure	Group L (n = 21)	Group R (n = 21)	*p*
**Age (years)**	70.4 ± 11.6	62.1 ± 18.4	0.088
**Sex (male/female)**	11/10	12/9	0.757
**Processing time (minutes)**	46.6 ± 11.4	43.9 ± 10.5	0.428
**Need for additional dose (yes/no)**	3/18	2/19	0.634
**VAS Score, median (Q1–Q3)**	4.0 (2.5–7.5)	1.0 (0–2.0)	<0.001 *
**MRC Muscle Power Scale**	5.0 ± 0	3.3 ± 1.0	<0.001 *
**PSPSQ QUESTION 1**	4.7 ± 1.6	6.7 ± 0.5	<0.001 *
**PSPSQ QUESTION 2**	4.1 ± 1.7	6.7 ± 0.5	<0.001 *
**PSPSQ QUESTION 3**	4.4 ± 1.9	6.8 ± 0.6	<0.001 *

Abbreviations. Group L: local anesthesia; Group R: regional anesthesia; VAS: Visual Analog Scale; PSPSQ: Post-Surgical Patient Satisfaction Questionnaire. Data are presented as mean ± standard deviation (SD) or median (Q1–Q3). * Statistical significance was set at *p* < 0.05. VAS assesses pain, while the MRC Muscle Power Scale rates strength from 0 (no contraction) to 5 (normal power). PSPSQ Q1–Q3 represent patient satisfaction, covering overall experience, pain management, and recovery.

## Data Availability

The datasets used in the current study are available from the corresponding author upon reasonable request.
